# A measure of regularity for polygonal mosaics in biological systems

**DOI:** 10.1186/s12976-015-0022-1

**Published:** 2015-11-16

**Authors:** Gabriela Contreras-Figueroa, Luis Hernández-Sandoval, José L. Aragón

**Affiliations:** Facultad de Ciencias Naturales, Universidad Autónoma de Querétaro, Av. de las Ciencias s/n, Juriquilla, Querétaro, 76230 Mexico; Centro de Física Aplicada y Tecnología Avanzada, Universidad Nacional Autónoma de Mexico, Boulevard Juriquilla 3001, Juriquilla, Querétaro, 76230 Mexico

**Keywords:** Regularity, Eutactic stars, Spatial patterns

## Abstract

**Background:**

The quantification of the spatial order of biological patterns or mosaics provides useful information as many properties are determined by the spatial distribution of their constituent elements. These are usually characterised by methods based on nearest neighbours distances, by the number of sides of cells, or by angles defined by the adjacent cells.

**Methods:**

A measure of regularity in polygonal mosaics of different kinds in biological systems is proposed. It is based on the condition of *eutacticity*, expressed in terms of *eutactic stars*, which is closely related to regularity of polytopes. Thus it constitutes a natural measure of regularity. The proposed measure is tested with numerical and real data. Numerically is tested with a hexagonal lattice that is distorted progressively and with a non-periodic regular tiling. With real data, the distribution of oak trees in forests from three locations in the State of Querétaro, Mexico, and the spiral pattern of florets in a flowering plant are characterised.

**Results:**

The proposed measure performs well and as expected while tested with a numerical experiment, as well as when applied to a known non-periodic tiling of the plane. Concerning real data, the measure is sensitive to the degree of perturbation observed in the distribution of oak trees and detects high regularity in a phyllotactic pattern studied.

**Conclusions:**

The measure here proposed has a clear geometrical meaning, establishing what regularity means, and constitute an advantageous general purposes alternative to analyse spatial distributions, capable to indicate the degree of regularity of a mosaic or an array of points.

## Background

Patterns, in time or space, play a central role in biological systems. Natural patterns are ubiquitous and are usually constructed by the spatial distribution of the constitutive elements of a particular system. Albeit pattern can not be defined rigorously, its geometric features “recur recognisably and regularly, if not identically or symmetrically” [1]. Particularly interesting patterns are the mosaics, where the constitutive elements are spatially arranged in a regular manner. Typical examples are the arrangement of retinal ganglion cells in mammals [2], arrangements of epithelial cells, and spatial arrangements of plant organs and cells. Closely related patterns are the ones formed by the arrangements or distributions of points in a plane; a typical example is the pattern of distribution of plant populations, which is of interest for ecologists since constitutes a fundamental characteristic of that population. In this case the corresponding polygonal pattern is obtained by the Voronoi tessellation associated to the distribution of points^1^.

The characterisation of patterns provide useful information. Ganglion cells in the mammal retina, for instance, are distributed in an economical way such that the visual field is optimally mapped [2]. Also, the adjacency of cells (called cell sociology by Chandebois [3]) is important for cell communication and signalling. Consequently the geometrical characterisation of mosaics and particularly its degree of regularity is an important issue in biological systems.

Among the existing methods to quantify the degree of regularity or poligonality of spatial patterns are those based on counting the number of neighbouring cells [4] and cell areas [5]. Recurring methods to study patterns of points are based on the distances between nearest-neighbours (NND) [6, 7]. The poligonality index [8] was more recently introduced and measures how far a tested polygon is from a regular polygon with an interior angle *β*. Both methods will be described in Section “Existing methods”.

In this work, we propose a measure of the regularity of a spatial pattern based on the condition of eutacticity, expressed in terms of eutactic stars. As we will see, the main advantage of this measure is that eutactic stars are closely related to regular polytopes thus it constitutes a natural measure of regularity, supported by geometrical meaning. The proposed measure was tested with numerical and real data. Numerically, it was first tested with a numerical experiment consisting of a progressive distortion of an hexagonal pattern and then it was applied to measure the regularity of a Penrose tiling, a well known non-periodic tiling of the plane with many interesting geometrical properties. The resulting measurements of regularity were compared with some existing methods. In real data, we use the proposed measure to characterise the distribution of points defined by the positions of *Quercus* individuals at three locations in the State of Querétaro, Mexico, and to characterise a phyllotactic spiral pattern. From the obtained results, we conclude that the measure based on eutacticity constitutes an advantageous general purposes alternative for analysing spatial distributions. It provides a single number indicating the degree of regularity of the studied pattern, independently of the number of sides of the polygons that compose the pattern. Also, the measure can be generalised in a straightforward way to study a three-dimensional distribution of points or a polyhedral pattern.

This paper is organised as follows. In Section “Mathematical background”, a mathematical background necessary to describe existing methods and to introduce our measure of regularity is presented. In Section “Results”, the proposed measured is applied to numerical and real data. Finally Section “Discussion” is devoted to discussion and conclusions.

## Mathematical background

### Existing methods

The NDD is a statistical method originally proposed for measuring spatial distribution in plant populations and considers a population with *N* individuals distributed in an area *A* with density *ρ*; if $ \langle r \rangle _{A} = \frac {\sum r_{i}}{N}$ is the average distances between nearest neighbours, where *r*_*i*_ is the distance from the individual *i* to its nearest neighbour, then it can be shown [6] that for a random spatial distribution of individuals the expected value of 〈*r*〉_*A*_ is $\langle r \rangle _{E} = \frac {1}{2\sqrt {\rho }}$. Therefore, the ratio

(1) $$\begin{array}{@{}rcl@{}} R = \frac{\langle r \rangle_{A}}{\langle r \rangle_{E}} \end{array} $$

measures the degree to which the distribution approaches or departs from a random one. *R* ranges from *R*=1 for a perfectly random distribution, *R*=0 for a completely aggregation to *R*=2.1491 for an hexagonal pattern. The null hypothesis is a random pattern and using the *p*-value, it is accepted for *p*>0.05 according to the *PAST* software [9] used in this work. The ratio of the mean NND to the standard deviation of the NDD has been called *conformity ratio* and is also a commonly used measure [7]. A comparison of some methods based on nearest-neighbour distances is presented in Ref. [10].

Considering the Voronoi tessellation of a set of points, the poligonality index [8] measures how far a tested polygon is from a regular polygon with interior angle *β* (=60° for an hexagon). That is, the measure considers how far each of the interior angles of the polygons forming the Voronoi tessellation is from *β*. For this purpose, given the Voronoi cell of a test point *k*, starting at an arbitrary neighbour, the *N*_*k*_ successive neighbours are determined as one turns around its Voronoi cell in a clockwise fashion and the angles *α*_*i*_ defined by the adjacencies are stored. Then the poligonality index of the test point is defined as [8]:

(2) $$\begin{array}{@{}rcl@{}} \triangle_{\alpha} \left(k \right) = \frac{1}{\sum_{i=1}^{N_{k}} \left|\alpha_{i} - \beta \right|+1}. \end{array} $$

This value ranges from 1, for perfect poligonality, to 0 for a lack of spatial order. If *β*=60°, the measure is called hexagonality index.

### Eutactic stars

The notion of *eutaxy* (from Greek *eu*=good and *taxy*=arrangement) is closely related with regularity. The condition of *eutacticity* is expressed in terms of *eutactic stars* and thus to explain the relationship with regularity we should first define what a star is. A *star* in a *n*-dimensional space $\mathbb {R}^{n}$ is a set of *M* vectors {**u**_1_,**u**_2_,…**u**_*M*_} with a common origin and *M*>*n*. Thus, given a star, it is *eutactic* if literally it is well arranged or orderly disposed. This fuzzy definition can be formalised by considering projections from higher-dimensional spaces, as follows. A star of *M* vectors in $\mathbb {R}^{n}$ (where *M*>*n*) is eutactic if it can be viewed as the projection of *M* orthogonal vectors in a *M*-dimensional space $\mathbb {R}^{M}$. More formally, if *P* is a projector from $\mathbb {R}^{M}$ onto $\mathbb {R}^{n}$, then a star *S*={**u**_1_,**u**_2_,…**u**_*M*_} in $\mathbb {R}^{n}$ is eutactic if there exist orthogonal vectors {**U**_1_,**U**_2_,…**U**_*M*_} in $\mathbb {R}^{M}$ such that **u**_*i*_=*P*(**U**_*i*_), for *i*=1,2,…,*M*. The connection with regularity arises in the field of regular polytopes as follows. A polytope is the general term of the sequence “point, line segment, polygon, polyhedron,...,” [11]. Thus, a polytope is the generalisation to higher dimensions of geometric objects such as polygon (in two dimensions) or polyhedron (in three dimensions) and consequently a regular polytope is a generalisation of the regular polyhedra (Platonic solids) to an arbitrary dimension. Concerning eutaxy, the swiss mathematician L. Schläfli [12] (who coined the name eutactic) proved that the vectors from the centre of any regular polytope to its vertices form an eutactic star. This fact establishes the relationship between eutaxy and regularity: the star associated to a regular polytope is eutactic (the inverse is not necessarily true: an eutactic stars is not necessarily associated to a regular polytope).

The definition of eutactic stars as projections from orthogonal vectors in a higher dimensional space can be put in a more practical form. This is achieved in a theorem due to Hadwiger [13]; let *S*={**u**_1_,**u**_2_,…**u**_*M*_} be a star in $\mathbb {R}^{n}$ and let $\mathbb {A}$ be the *n*×*M* matrix whose *i*-th column is formed by the components of **u**_*i*_ with respect to the standard basis of $\mathbb {R}^{n}$. The star *S* is eutactic if and only if

(3) $$\begin{array}{@{}rcl@{}} \mathbb{A} \mathbb{A}^{T} = \lambda\mathbb{I}, \end{array} $$

for some real number *λ*. Here $\mathbb {A}^{T}$ denotes the transpose of the matrix $\mathbb {A}$ and $\mathbb {I}$ is the *n*×*n* identity matrix. When experimental measurements are involved, a more suitable criterion of eutacticity has been proposed [14]: if we define ${\mathbb {S}} = {\mathbb {A}} {\mathbb {A}}^{T}$ then the star producing $\mathbb {A}$ is eutactic if and only if

(4) $$\begin{array}{@{}rcl@{}} \varepsilon \equiv \frac{\text{Tr} \left({\mathbb{S}} \right)}{\sqrt{\text{Tr} \left({\mathbb{S}}{\mathbb{S}} \right)} \sqrt{n}}=1. \end{array} $$

Contrary to (3), criterion (4) has the advantage that the parameter *ε* is capable of indicating the degree of eutacticity of a star which is not strictly eutactic, because the closer this quantity is to 1, the more eutactic the star is. This property will be particularly useful in this work. Actually, it can be proved that the lower bound of *ε* is $1/\sqrt {n}$ [14]. Since in this work we will be concerned with polygonal patterns in the plane, stars are defined in two dimensions (*n*=2) and the lower bound of *ε* is in this particular case $1/\sqrt {2} \approx 0.7071$.

Eutactic stars have been particularly useful in several realms beyond the field of regular polytopes and even it has also been observed that the stars associated with the five ocular plates of sea urchins are eutactic through geological time, with some rare exceptions [15]. In what follows, eutactic stars will be used to quantify the degree of regularity of a polygonal pattern.

## A measure or regularity based on eutacticity

The idea behind the use of eutactic stars to measure the regularity of polygonal patterns is quite simple. To any polygon of the pattern it can be associate a star formed by the vectors from the centre to the vertices of the polygon. A measure of the eutacticity of this star, using (4), is a measure of the regularity of this particular polygon. The regularity of the pattern can be then the average of the value of eutacticity of the stars associated with each polygon of the mosaic. In the case of point patterns, these can be associated to polygonal patterns using Voronoi tessellations. In general, we can summarise the procedure as follows.

1. Given a pattern of points, its Voronoi partition is calculated.
2. Consider the set of all polygons of the Voronoi partition.
3. To avoid non-representative polygons, remove from this set all polygons with at least one vertex outside the convex hull. Let *Σ* be this set of representative polygons and assume that it contains *N* elements.
4. Now consider the set containing a measure of the eutacticity of each polygon in *Σ*, that is, from (4), {*ε*_1_,*ε*_2_,…*ε*_*N*_}.
5. An estimation of the regularity of the tessellation is $\langle \varepsilon \rangle = \frac {1}{N} \sum _{i=1}^{N} \varepsilon _{i}$.

Since the above measure averages the eutacticity values of the stars associated with each polygon of the pattern, the presence of large voids of clustering can not be detected. This can be alleviated by considering the dispersion on sizes of the vectors forming the stars, since stars at the neighbourhood of a cluster should have larger size dispersion. Thus, consider a star *S*={**u**_1_,**u**_2_,…,**u**_*N*_}. If *u*_*i*_=∥**u**_*i*_∥, where ∥ ∥ stands for the Euclidean norm, then the average size of the vectors belonging to the star *S* is $\langle u \rangle _{S} = \frac {1}{N} \sum _{i=1}^{N} u_{i}$, and the dispersion on size of these vectors can be calculated by means of the standard deviation:

$$\begin{array}{@{}rcl@{}} \sigma_{S} = \sqrt{\frac{1}{N} \sum\limits_{i=1}^{N} \left(u_{i}- \langle u \rangle_{S} \right)^{2}}. \end{array} $$

Then, for a polygonal array composed of *N* polygons, the following measure of regularity is proposed:

(5) $$\begin{array}{@{}rcl@{}} E = \left\langle \frac{\varepsilon}{1 + \frac{\sigma_{s}}{\langle u \rangle_{S}}} \right\rangle = \frac{1}{N} \sum\limits_{i=1}^{N} \frac{\varepsilon_{i}}{1 + \frac{\sigma_{s_{i}}}{\langle u \rangle_{S_{i}}}}. \end{array} $$

Notice that for tessellations composed by regular polygons *ε*_*i*_=1 and $\sigma _{\varepsilon _{i}} = 0$ for all *i*, thus *E*=1, which is the higher value of *E*. The lower bound of *ε* is $1/\sqrt {2}$ but the higher value of $\sigma _{\varepsilon _{i}}$ depends on the dispersions on size of the vectors composing the star; the more clustered the tessellation, the larger $\sigma _{\varepsilon _{i}}$ and, consequently, the smaller *E*.

We should emphasise that the measure (5) is designed to indicate whether a mosaic is regular (*E*=1) or not (*E*≠1) but the closer this value is to one, the more regular a mosaic is.

## Results

### Numerical data

The measure (5) is invariant under translations, rotations or scalings, as can be inferred from (4) and (5) itself. The behaviour and sensitivity of the proposed measure can be tested by applying a numerical experiment proposed in [8]. As starting point, an hexagonal lattice composed by 661 vertices with edge length equal to 1 is generated. Now, controlled distortions of this structure are applied by randomly picking 200 vertices and each one is translated by a vector with magnitude *δ* pointing to a random direction. The resulting set of points were analysed by applying the proposed measure (5) as well as NND (1) and the hexagonality index (2). The magnitude of the perturbation was varied from 0 to 1.2 in steps of *δ*=0.1.

**Fig. 1:**
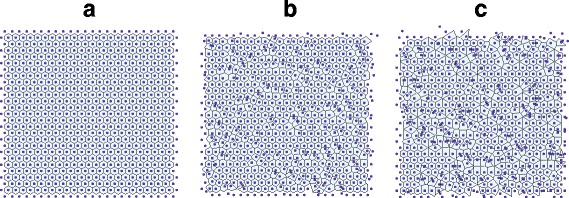
Some examples of the numerical experiment that consists of perturbing an hexagonal lattice, as described in the text, with perturbation magnitudes *δ*=0.0 (**a**), *δ*=0.5 (**b**), and *δ*=1.0 (**c**)

**Fig. 2:**
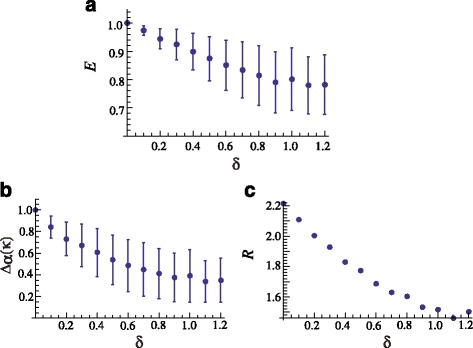
Regularity measurements versus the magnitude of the perturbation *δ*. **a** Measure proposed in this work; **b** Hexagonality index; **c** NND

In Fig. 1m, the original hexagonal lattice (with dots) and its Voronoi tessellation is shown. Two examples of perturbed lattices, with *δ*=0.5 and *δ*=1.0, are shown in Figs. 1b and c, respectively. The behaviour of *E*, *R* (NND) and △_*α*_(*k*) (hexagonality index) versus *δ* are shown in Fig. 2. All the measurements display the gradual decreasing of regularity expected with the progressive perturbations and both the eutacticity and the hexagonality index behave linearly with the progressive perturbations. The values of eutacticity range from *E*=1 for non perturbed data to *E*=0.7824 for *δ*=1.2. Similarly, the hexagonality index ranges between △_*α*_(*k*)=1, for no perturbations, to △_*α*_(*k*)=0.53504 for the maximum perturbation. The NND index behaves also linearly and indicates an over-dispersed or regular arrangement since values are larger than *R*=1, with *p*<0.05.

Notice that the behaviour of the three measures is similar and some comments about this similarity can be said here. Despite that the NDD measure (*R*) shown in Fig. 2c show a continuous decreasing, it can not be said that the regularity of the pattern is decreasing accordingly. As it was mentioned in Section “Existing methods”, the NDD method was designed to statistically differentiate between random, aggregate and over-dispersed (regular) distributions; besides the *p* value, that verifies whether the null hypothesis is fulfilled or not, the departure from regularity or randomness must be asserted with a significance test [6]. Concerning the hexagonality index, this continuous decreasing of the regularity is expected since the numerical test consists of the controlled distortion of an hexagonal array and even with the largest distortion factor applied (*δ*=1.2), 47.5 % of the polygons have six edges, that is, distorted hexagons, against 21.5 % with five, 16.6 % with seven, 7.5 % with four, 5 % with eight, 0.8 % with three, 0.6 % with nine and 0.5 % with ten edges. A different scenario may arise if polygons with different number of edges are distributed more or less equally, as we will see in what follows.

**Fig. 3:**
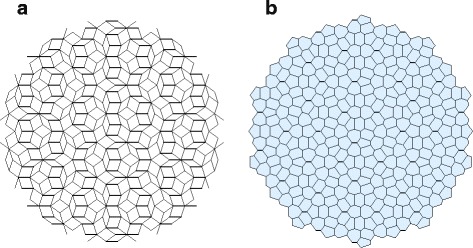
Fragment of a Penrose tiling (**a**) and its Voronoi tessellation (**b**)

One of the most remarkable tiling of the plane is the so-called Penrose tiling. It has pentagonal symmetry, it is non periodic but with long-range order and exceptional geometric properties (for a general reference on Penrose tilings see Ref. [16]). Albeit no examples of this kind of aperiodic patterns have been observed in a biological system, it is interesting to apply the measures to this ideal structure. In Fig. 3 a fragment of a Penrose tiling obtained by the dual generalised method [17] and its respective Voronoi tessellation (resembling a cellular structure) is shown. The distribution of pentagons, hexagons and heptagons in this Voronoi tessellation is, respectively, 31.4 %, 39.2 % and 29.4 %. The measure based on eutacticity (5) yields *E*=0.903, indicating that the pattern is highly regular, whereas the hexagonality index (2) gives △_*α*_(*k*)=0.443, which is too low for a pattern, which is known to be regular. The NND index (1) gives *R*=1.729, indicating that the pattern is regularly distributed.

### Application to real data

#### The distribution of oak trees

Albeit the proposed measured (5) can be used to characterise general mosaic or point patterns in any biological systems, it is illustrative to study the spatial distribution of plants, which is important to understand the dynamics of the ecosystem of plant communities, as well as the morphological and environmental factors that produce a particular spatial pattern [18]. The NND measure has been applied to this problem but more specific measures has been developed for this particular problem in ecology, namely, the Ripley’s K-function [19] and the Spatial Analysis by Distance Indices (SADIE) [20]. In these methods the position of the plant defines a point in the plane, the null model is a completely random distribution of points and the departure from the null model yields two alternatives. In the first one, there is a high probability of finding points close together and the patterns is called aggregated, clumpy or clustered. On the contrary, in the second case, for a given point there is a low probability of finding points close to it and this pattern is called over-dispersed or regular.

We used (5) to study the distribution of trees in localities with environmental influence. Field data was acquired by sampling three oak forest in the state of Querétaro, Mexico:

1. Laguna de Servín, Amealco de Bonfil (20° 15^′^48^″^*N*, 100° 15^′^23^″^*W*).

2. Escolásticas, Huimilpan (20° 24^′^57^″^*N*, 100° 15^′^49^″^*W*).

3. Xajay, Amealco de Bonfil (20° 03^′^20^″^*N*, 99° 58^′^02^″^*W*).

Forests with individuals of the genus *Quercus* are called Oak forests. Despite that in the same forest could coexist different *Quercus* species, no distinction between species was done on the sampled sites so that an univariate (single-species) analysis was performed.

Using a *Magellan-ProMark 3* GPS with millimetre resolution, the trunk’s position of each tree was registered and mapped onto a plane. The circumference at breast height (CBH) of each trunk was registered as well as the number of damaged or destroyed trees.

The three sampled locations turned out to be different, concerning its level of preservation. At Laguna de Servín, 181 individuals were mapped in a relatively flat area of 1576 m^2^. Laguna de Servín is located behind a road, showing human deforestation (27 % of sampled individuals were damaged). At Escolásticas, 115 trees in an area of 11 635 m^2^ were sampled and despite that this location is a oak patch near to pasture and roads, it was the less perturbed since only 3 % of sampled individuals were injured. At Xajay 195 individuals within an area of 3 350 m^2^ were sampled. Even that Xajay is located on a top hill (3000 masl), human deforestation is common around the zone and about 4 % of the trees were cut down. The measured CBH average values were 75 cm for Laguna de Servín, 160 cm for Escolásticas and 90.5 cm for Xajay.

**Fig. 4:**
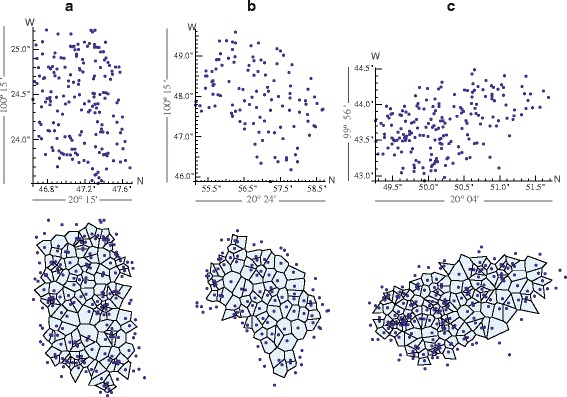
Oak forest sampled in three locations of the state of Querétaro, Mexico. The information of each forest is arranged in columns: **a** Laguna de Servín, **b** Escolásticas and **c** Xajay. Their respective Voronoi tessellations are shown below each one. Each point correspond to the trunk of a mapped tree and the (*x,y*) values are given in geographic coordinates

The mapped points for each location is shown in Fig. 4. Plots are arranged in three columns corresponding to each location: (a) Laguna de Servín, (b) Escolásticas, and (c) Xajay. In each column, the mapped points are shown on top and its respective Voronoi diagram at the bottom. Each set of points was analysed using the measures (5), (2) and (1), and the results are shown in Table 1. Notice that here also the three measures yield similar results: the highest value of regularity is obtained in Escolásticas, followed by Xajay and Laguna de Servín. With the exception of Laguna de Servín, where *p*>0.05, indicating a random distribution (despite that *R*>1), the NND measure indicates that in Xajay and Escolásticas the distribution is regular but the significance of the departure must be tested statistically. The eutacticity criterion and the hexagonality index were sensitive to the degree of perturbation observed. It should be said however that the Voronoi tessellation of the distribution of trees in Escolásticas, shown in Fig. 4, contains 44.6 % of polygons with six sides, against 21.6 % with five, 19 % with seven, 10.8 % with four and 4 % with eight sides. That is, most of the polygons have six sides, a fact that favours the hexagonality index.

**Table 1:** Results of the spatial analysis applied to the three forests sampled

Location	*E*	*Δ*_*α*_(*k*)	*R*
Laguna de Servín	0.6863	0.2095	1.058 (*p*>0.05)
Escolásticas	0.7423	0.2355	1.1624 (*p*<0.05)
Xajay	0.6926	0.2050	1.0805 (*p*<0.05)

The regularity measured in Escolásticas can be also interpreted by taking into account that this locality has the largest average value of the CBH. Ecologically, it indicates the presence of long-lived trees that have reached a considerable size to divide the space among its neighbours in an homogeneous way. Additionally, Escolásticas is the locality with the less perturbation, yielding a higher value of regularity.

#### A spiral phyllotactic pattern

**Fig. 5:**
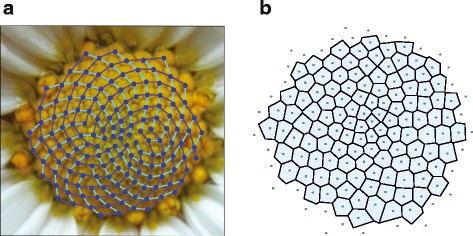
**a** Digital image of a flowering plant of the species *Leucanthemum maximum*, showing the spiral pattern of florets (spirals are drawn on top as a guide to the eye). **b** Voronoi tessellation associated to the set of points defined by the florets

The arrangement of plant organs, also called phyllotaxis, has fascinated scientists and naturalists for centuries, mainly because it is dominated by remarkable mathematical relationships. Among the many different types of plant organs arrangements, perhaps the most conspicuous and complex is the spiral pattern as in sunflowers. Since the 19^th^ Century, it was known the relation between the Fibonacci sequence and the phyllotactic spirals (for a general reference see (Ref. [21] Ch.4)): the number of spirals (parastichies) are generally consecutive numbers of the series 1,1,2,3,5,8,13,…, which is the Fibonacci series, each of whose terms is the sum of the preceding two. The Fibonacci sequence is closely related with the golden mean $\tau = \left (1 + \sqrt {5} \right) / 2$, since if *F*_*n*_ is the *n*-th term of the Fibonacci sequence, then *F*_*n*+1_/*F*_*n*_→*τ* in the limit *n*→*∞*. Interestingly, the Penrose tiling, as the shown in Fig. 3a, and the golden mean are closely related since the ratio between the diagonal and the side of a pentagon is *τ*. In Fig. 5a the spiral phyllotaxis of a flowering plant of the species *Leucanthemum maximum* is shown, where the spirals have been drawn as a guide to the eye. The coordinates of each floret where found from the digital image using the morphometric software package *Image J* [22] and in this way a distributions of points in a plane was obtained, whose Voronoi tessellation is shown in Fig. 5b. The regularity of this tessellation was quantified using the measures (5), (2), and (1). The measure based on the eutacticity yields *E*=0.9114, as expected from a highly regular pattern, whereas the hexagonality index (2) gives △_*α*_(*k*)=0.4956, which is again too low for a regular pattern. The NND index (1) gives *R*=1.8693, indicating that the pattern is regularly distributed.

## Discussion

The condition of eutacticity offers a formal definition of regularity in geometric forms that can be associated with a star of vectors. This fact is used to measure the regularity of a polygonal array or a set of points since a star of vectors can be associated with each polygon of the pattern or with the Voronoi polygon of a point. The measure thus proposed has a clear geometrical meaning and constitutes a general purposes natural way to measure regularity.

The measure is tested with numerical data by means of a numerical experiment consisting of a progressive distortion of a hexagonal pattern and it was also applied to the set of vertices of a Penrose tiling, a well known non-periodic pattern of the plane. In the former case, the measure behaved as expected and was able to detect small perturbations. It should be said that the hexagonality index behaves also well and is equally sensitive as was already reported in [8] but this could be expected since the distorted array is an hexagonal one. When applied to the Penrose tiling, the proposed measure yields that the tiling is highly regular, as expected, whereas the hexagonality index is not able to detect this regularity since the polygonal array is composed by pentagons, hexagons and heptagons, in almost the same percentage. In both cases, the NND measure performs well. We should say however that this measure is useful in ecology for the specific purpose of detecting random, clustered or regularly arranged patterns, then it has a statistical basis instead of a geometrical one, as the measure proposed here. The quantification of the departures from regularity or randomness, for instance, requires a significance test.

Concerning the application to the real data of distribution of trees in three oak forest in the state of Querétaro, Mexico, some comments are as follows. Albeit as mentioned in Section “Results” specific measures has been developed for this particular problem in ecology, the proposed measure was capable to detect irregularities that seems to be related with the level of the forests preservation. The hexagonality index was also capable to detect these irregularities but, as mentioned in Section “Application to real data”, the Voronoi tessellation associated with the most preserved forest turned out to be the one with most polygons with six edges. Contrary to the measures proposed for ecological problems, as the NND, the measure based on eutacticity is not capable to discern between over-dispersed and clustered distribution, which is of interest to ecologists. We should say however that in general the spatial order is not necessary defined by clustered, over-dispersed or random aggregations. The measure proposed here gives a geometric alternative with a range of values that indicate if a mosaic or an array of points is more or less regular based on geometrical concepts. The combination of this measure with statistical methods already proposed would bring complementary information about the space availability for plant and its neighbours. Finally, when applied to a phyllotactic spiral pattern, the proposed measure detected a high regularity, as expected.

The measure (5) then is capable to detect regular patterns and to provide a measure of the regularity. This makes the measure useful in several realms where star of vectors can be defined, for instance to detect regularities in complex networks [23, 24], where links emanating from a node define a vector star.

An additional advantage of the proposed measure, is that can be easily generalised to study a three-dimensional distribution of points or a polyhedral pattern, by considering three-dimensional vector stars. Actually in this case it is enough to set *n*=3 in Eq. (4).

## Conclusions

In this work we propose a general purposes measure of regularity in polygonal mosaics or point patterns in biological systems. It assigns a single value to the mosaic or the collection of points in the plane indicating its degree of regularity.

The measure performs well and as expected while tested with numerical data. In an example with real distribution of oak trees, the measure is sensitive to the degree of perturbation observed, which produces a less regular distribution of trees. In a second example with real data, the measure is capable to detect the high regularity of a phyllotactic spiral pattern.

The main advantage of the proposed measure over other methods used for this purposes is that it has a clear geometrical meaning since the condition of eutacticity rests on the property of regularity, thus constitutes a natural way to measure regularity, which is independent of the type of polygons that form the pattern. It has also the advantage that can be used for studying three-dimensional distribution of points or polyhedral patterns. The measure for regularity here proposed has a clear geometrical meaning and constitutes an alternative for analysing spatial distributions in different systems or arrays, capable to indicate the degree of regularity of a mosaic or an array of points.

## Methods

A measure for regularity for polygonal mosaics of different kinds in biological systems is proposed. It is base on the condition of eutacticity, expressed in terms of eutactic stars, which is closely related to regular polytopes. All the input data consisted of the (x, y) coordinates of sets of points coming from two sources: numerically generated and measured from real data. In both cases these coordinates were handled with Wolfram Mathematica 10.3 [26]. Voronoi tessellations and convex hulls were calculated with the built-in functions included in the Computational Geometry Package, now built into the Wolfram System. The procedure to associate a star vector to each Voronoi polygon, to calculate its value of eutaciticy and the numerical algorithm described in Section “Numerical data” were all codified as user-defined Mathematica functions. The hexagonality index was also codified as a Mathematica function. The value of NDD for a given set of points (numerically generated or measured) was obtained with the PAST software [9]. Concerning real data, the (x,y) coordinates of the trunk’s positions of oak trees were obtained by means of a Magellan-pro Mark 3 GPS with millimetre resolution; these coordinates were stored in a file, which was after imported into Mathematica. The (x,y) coordinates of the florets in the studied phyllotactic pattern were found from the digital image of the flowering plant using the morphogenetic package ImageJ [22] and the resulting file was then imported into Mathematica.

## Endnote

^1^ For an introduction to Voronoi tessellation’s see for instance (Ref. [25] Ch. 2).
